# A convergent subcortical signature to explain the common efficacy of subthalamic and pallidal deep brain stimulation

**DOI:** 10.1093/braincomms/fcad033

**Published:** 2023-02-12

**Authors:** Leon A Steiner, Luka Milosevic

**Affiliations:** Krembil Brain Institute, University Health Network, Toronto M5T 2S8, Canada; Department of Neurology, Charité-Universitätsmedizin Berlin, Berlin 10117, Germany; Berlin Institute of Health (BIH), Berlin 10178, Germany; Krembil Brain Institute, University Health Network, Toronto M5T 2S8, Canada; KITE Research Institute, University Health Network, Toronto M5G 2A2, Canada; Center for Advancing Neurotechnological Innovation to Application (CRANIA), Toronto M5T 2S8, Canada; Institute of Medical Sciences, University of Toronto, Toronto M5S 1A8, Canada; Institute of Biomedical Engineering, University of Toronto, Toronto M5S 3G9, Canada

## Abstract

This scientific commentary refers to ‘Globus pallidus internus deep brain stimulation evokes resonant neural activity in Parkinson’s disease’, by Johnson *et al*. (https://doi.org/10.1093/braincomms/fcad025).

**This scientific commentary refers to ‘Globus pallidus internus deep brain stimulation evokes resonant neural activity in Parkinson’s disease’, by Johnson *et al*. (https://doi.org/10.1093/braincomms/fcad025).**

We have read with great interest the article by Johnson and colleagues^1^ describing novel observations of *pallidal* evoked resonant neural activity (ERNA) during deep brain stimulation (DBS) of the globus pallidus internus (GPi).

ERNA has garnered much research interest following its description by Sinclair *et al*. in 2018 as an electrophysiological signature which occurs during DBS of the subthalamic nucleus (STN).^2^ This phenomenon was subsequently reproduced by colleagues across various DBS centres,^3–5^ and it has moreover been reported that recordings of ERNA are of greatest amplitude at contacts that produce the best clinical effects when used for stimulation.^2,6^ Such findings suggest that ERNA could be considered as a physiomarker to guide DBS implantation location and titration of stimulation settings, as now corroborated by Johnson *et al*.^1^ in the context of GPi-DBS. Importantly, the neural basis of ERNA has been validated experimentally, to rule out the possibility that this signature is a stimulation-induced artefact.^7^

Our own group has also become interested in further corroborating the single-neuron basis of this stimulation-induced electrophysiological signal in the context of microelectrode stimulation and recordings acquired during awake DBS implantation surgery. These recordings allow for the concurrent acquisition of stimulus-evoked field potentials and associated changes to neuronal spiking during stimulation delivery. In this context, we have reproduced observations of STN ERNA, and have demonstrated that the ERNA waveform is associated with temporally-locked patterned neuronal inhibition (Steiner *et al*., *in preparation*). An example of this phenomenon from a single STN recording site during 100 Hz stimulation is available in Fig. 1A. Mechanistically, we have proposed that positive-going *extracellular* field potentials are mediated by stimulation-induced activation of GABAergic inputs to STN (homologous to *intracellular* inhibitory postsynaptic potentials), originating from the globus pallidus externus (GPe).^8^ As such, the initial peak of the STN ERNA waveform is likely the result of the direct activation of the afferent inputs to STN, producing an inhibitory net response that is predominantly mediated by GPe. Concurrently, activation of STN efferent axons can be expected to cause downstream release of glutamate at STN-GPe synapses. The excitation of GPe would thereafter result in recurrent inhibition to STN, producing the evoked resonant phenomenon, as first proposed by Schmidt *et al*.^5^ and summarized in Fig. 1A.

**Figure 1:**
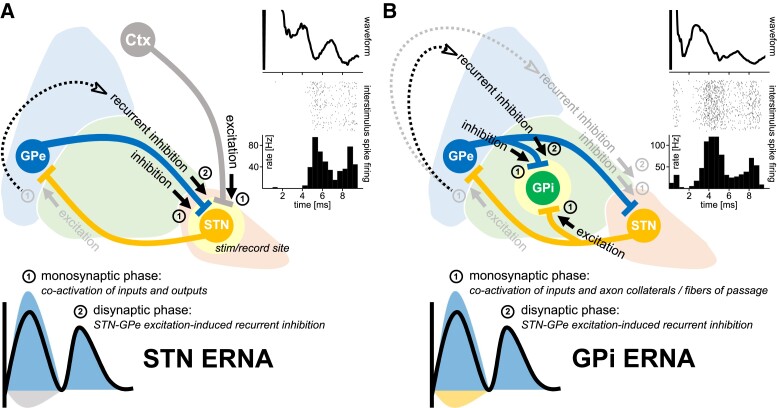
**(A)** STN and **(B)** GPi ERNA. Inserts in the top right of each figure are intraoperative ERNA waveforms (averaged across successive stimuli at 100 Hz), spike rater plots, and peristimulus histograms. These figures demonstrate that ERNA waveform peaks are associated with time-locked neuronal inhibition. Schematics depict hypotheses as to how ERNA may emerge in (**A**) STN and (**B**) GPi. In each case, the emergence of ERNA is hypothesized to be dependent on activation of the STN-GPe excitatory-inhibitory network.

Inspired by the work of Johnson *et al*.,^1^ our group has also become interested in understanding how GPi-DBS may give rise to ERNA. Firstly, it is important to qualify that we have indeed reproduced observations of GPi ERNA in the intraoperative context. An example of one recording site in GPi is shown in Fig. 1B. Like in STN, the GPi ERNA waveform appears to be associated with temporally patterned neuronal inhibition. Our hypothesized circuit activation profile for GPi ERNA is summarized in Fig. 1B. Similar to STN ERNA, we propose that the initial peak is a result of direct activation of afferent inputs to GPi, producing a net-inhibitory response. Concurrent to the activation of afferent inputs, GPi-DBS may invade the axon collaterals^9^ of afferent inputs, as well as activate fibres of passage, leading to neurotransmitter release at remote sites (i.e. release of glutamate in GPe and GABA in STN). Following this principle, the invaded STN-GPe projections would result in the excitation of GPe, which would thereafter lead to recurrent inhibition of GPi, and therefore, ERNA.

Although the term *ERNA* was first established by Sinclair *et al*.,^2^ a 2003 study by Hashimoto and colleagues from the group of Jerrold Vitek showed that STN-DBS also lead to highly patterned interstimulus spiking in the GPe.^10^ However, the patterned interstimulus spiking in GPe was in fact elevated above baseline (i.e. *excitatory* responses) and temporally shifted when compared to the suppressed interstimulus spiking patterns (i.e. *inhibitory* responses) we have observed STN and GPi. Together, these observations provide support for the hypothesis of a reciprocal excitatory-inhibitory network activation phenomenon. In addition to the co-occurrence of (phase-shifted) ERNA patterns in STN and GPe during STN-DBS, it would be reasonable to expect the co-occurrence of ERNA in GPi, which has indeed been demonstrated by Schmidt and colleagues.^5^ By the same logic, and as capitulated in schematic Fig. 1B, ERNA would be expected to co-occur in STN during GPi-DBS. To this end, it has been shown that effective GPi-DBS suppressed STN neuronal firing, hypothesized to be mediated by activation of GPe-STN fibres.^11^ As such, ERNA seems to be representative of an electrophysiological signature common to multiple nodes of the basal ganglia, which can be directly initiated by STN-DBS or indirectly through invasion of this circuitry by GPi-DBS, and appears to be orchestrated by the reciprocal excitatory-inhibitory connectivity of the STN-GPe loop.

These multi-nodal observations of ERNA are perhaps important for answering a long-standing DBS research question: is there a convergent mechanistic theory to explain the common efficacy of STN- and GPi-DBS in Parkinson’s disease? Some have postulated that the beneficial effects of STN-DBS are mediated by antidromic activation of the cortico-STN hyperdirect pathway fibres. However, as demonstrated in a non-human primate study by the Vitek group, this same phenomenon is not present during GPi-DBS.^12^ Recent work from the group of Andreas Horn has suggested an overlap in the cortical functional (fMRI-based) connectomic profiles associated with STN- and GPi-DBS.^13^ However, the question remains: how might this common therapeutically discriminative cortical network signature arise? We hypothesize that it is the result of a coordinated activation of *subcortical* circuitry, that can be evoked by therapeutically-relevant STN- *or* GPi-DBS (as now demonstrated), ultimately producing a common downstream modulatory effect upon the greater basal-ganglia-thalamo-cortical network.

## Data Availability

Data sharing is not applicable to this article as no new data were created or analysed.
